# Rheumatoid arthritis decreases risk for Parkinson’s disease: a Mendelian randomization study

**DOI:** 10.1038/s41531-021-00166-x

**Published:** 2021-03-02

**Authors:** ChunYu Li, RuWei Ou, HuiFang Shang

**Affiliations:** Department of Neurology, Laboratory of Neurodegenerative Disorders, National Clinical Research Center for Geriatric, West China Hospital, Sichuan University, Chengdu, China

**Keywords:** Parkinson's disease, Risk factors

## Abstract

Epidemiological and clinical studies have suggested comorbidity between rheumatoid arthritis and Parkinson’s disease (PD), but whether there exists a causal association and the effect direction of rheumatoid arthritis on PD is controversial and elusive. To evaluate the causal relationship, we first estimated the genetic correlation between rheumatoid arthritis and PD, and then performed a two-sample Mendelian randomization analysis based on summary statistics from large genome-wide association studies of rheumatoid arthritis (*N* = 47,580) and PD (*N* = 482,703). We identified negative and significant correlation between rheumatoid arthritis and PD (genetic correlation: −0.10, *P* = 0.0033). Meanwhile, one standard deviation increase in rheumatoid arthritis risk was associated with a lower risk of PD (OR: 0.904, 95% CI: 0.866–0.943, P: 2.95E–06). The result was robust under all sensitivity analyses. Our results provide evidence supporting a protective role of rheumatoid arthritis on PD. A deeper understanding of the inflammation and immune response is likely to elucidate the potential pathogenesis of PD and identify therapeutic targets for PD.

Parkinson’s disease (PD) is the second most common neurodegenerative disease, pathologically characterized by the progressive degeneration of dopaminergic neurons in the pars compacta of the substantia nigra^1^. The etiology of PD is multifarious and complex, with a sophisticated interaction between genetic and environmental factors^2^. Currently, compelling evidence implicates that inflammation and immunity play an important role in the pathogenesis of PD^3^, and the comorbidity between PD and autoimmune diseases has been reported^4^.

Rheumatoid arthritis is the most common autoimmune disorder, with a core feature of abnormal activation of inflammation and immunity. Although some cases of rheumatoid arthritis co-existing with PD have been reported^4^, the association between rheumatoid arthritis and PD remains elusive and controversial. Conventionally, rheumatoid arthritis was presumed to be associated with increased risk of PD, due to the overproduction of inflammatory mediators and overactivation of immune cells^5,6^. This hypothesis was further supported by studies from postmortem brain tissue of PD that demonstrated increased levels of proinflammatory mediators in the striatum of the brain^7^. However, recent cohort studies showed that patients with rheumatoid arthritis have a reduced risk of developing PD^8,9^, which was contrary to previous results. Nevertheless, such observational studies were influenced by the possibility of confounding factors, such as the use of nonsteroidal anti-inflammatory drugs (NSAIDs)^8^. The unadjusted confounding factors might bias the association evidence, as is difficult to avoid in observational studies. Therefore, whether rheumatoid arthritis affects the risk of PD remains unknown. Unraveling this association could help better understand the pathophysiology of PD and autoimmune diseases, and may facilitate the development of novel therapeutic targets.

Mendelian randomization (MR) is an advanced genetic epidemiological method, which is widely used to exploit the causal relationship between exposures and outcomes^10,11^. MR employs single nucleotide polymorphisms (SNPs) as instrumental variables for the exposure, thus it can effectively avoid the confounding factors from the change of related traits and has high scientific value. In order to decipher the potential causal relationship between rheumatoid arthritis and PD, a two-sample MR approach was employed to examine whether rheumatoid arthritis affects the risk of PD from a genetic perspective.

We first analyzed the genetic correlation between PD and rheumatoid arthritis, and detected a genome-wide significant and negative genetic correlation (genetic correlation: −0.10, *P* = 0.0033). Then we analyzed the role of rheumatoid arthritis on the risk of PD using the MR approach. As a result, each one standard deviation increase in rheumatoid arthritis risk was associated with a lower risk of PD (OR: 0.904, 95% CI: 0.866–0.943, P: 2.95E–06), and such results were supported using another three MR methods, namely MR–Egger regression (OR: 0.894, 95% CI: 0.836–0.957, P: 0.007), weighted mode (OR: 0.860, 95% CI: 0.805–0.918, P: 5.98E–04), and weighted median (OR: 0.915, 95% CI: 0.859–0.975, P: 0.006) (Fig. 1a, b). The funnel plot displays a symmetric pattern of effect size variation around the point estimate (Fig. 1c). The simple median method did not show a significant association but suggested the same direction of effect. The association was still significant after excluding variants within the MHC region, defined as base positions 24,000,000 to 35,000,000 on chromosome 6 (GRCh37) (OR: 0.920, 95% CI: 0.853–0.993, P: 0.031). We further replicated the results using exposures from another large GWAS on rheumatoid arthritis (OR: 0.929, 95% CI: 0.889–0.970, P: 8.60E–04) using the IVW method. And the association was significant after removing SNPs in the MHC region (OR: 0.930, 95% CI: 0.866–0.999, P: 0.047).

**Fig. 1:**
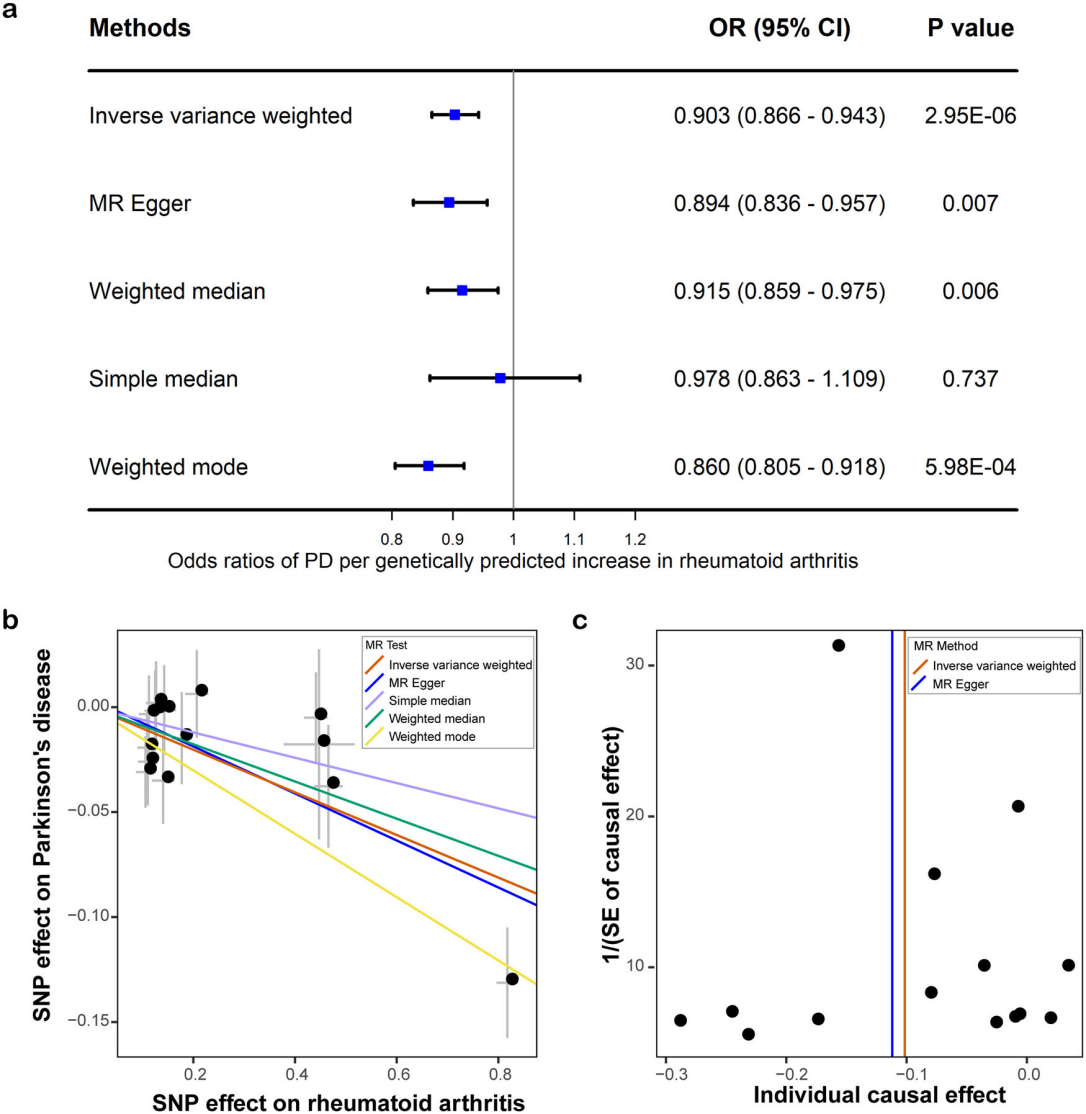
Mendelian randomization analysis results. **a** Forest plot showing results from the Mendelian randomization (MR) analysis to evaluate potential causal associations between rheumatoid arthritis and PD. Estimates are per 1 standard deviation increase in the trait. **b** Scatter plot of single nucleotide polymorphism (SNP) potential effects on rheumatoid arthritis versus PD. The 95% CI for the effect size on PD is shown as vertical lines, while the 95% CI for the effect size on rheumatoid arthritis is shown as horizontal lines. The slope of fitted lines represents the estimated Mendelian randomization effect per method. **c** Funnel plot for rheumatoid arthritis shows the estimation using the inverse of the standard error of the causal estimate with each individual SNP as a tool. The vertical line represents the estimated causal effect obtained using IVW and MR–Egger method.

Furthermore, we performed extensive sensitivity analyses to validate the association between rheumatoid arthritis and PD. We did not detect heterogeneity of effects based on Cochran’s Q test results (Table 1). The F statistics of all the instrument variables were high enough (ranging from 27 to 1581) to support the absence of weakness in the chosen instrument variables. Meanwhile, no apparent horizontal pleiotropy was detected as the intercept of MR–Egger was not significantly deviated from zero (Table 1). Violation of the causality was not found by directionality examination using Steiger analysis either. And we detected no potential instrumental outlier at the nominal significance level of 0.05 with MR-PRESSO analysis. The leave-one-out results suggested that the estimated causal effect was not driven by a single instrumental variable.

**Table 1:** Heterogeneity and horizontal pleiotropy analyses between rheumatoid arthritis and PD.

Exposure	Heterogeneity			Horizontal pleiotropy		
	IVW Q	IVW Q df	IVW P value	Egger intercept	SE	P
Discovery rheumatoid arthritis	13.58	13	0.40	3.64E–03	9.17E–03	0.70
Replication rheumatoid arthritis	10.48	7	0.16	−7.39E–04	1.66E–02	0.97

*IVW* Inverse variance weighted, *Q* Cochran’s Q test estimate, *df* Cochran’s Q test degrees of freedom, *SE* standard error.

In the past, various clinical studies have emerged to investigate the co-occurrence of rheumatoid arthritis and PD^4^. However, the relationship between rheumatoid arthritis and PD is still quite inconsistent and controversial^6,8,9^. For example, a population-based cohort study found that patients with rheumatoid arthritis had a significantly higher risk of developing PD (hazard ratio (HR): 1.14, 95% CI: 1.03–1.20)^6^. In contrast, another population-based case-control study from Denmark found that patients with rheumatoid arthritis have a decreased risk of developing PD (OR: 0.70, 95% CI: 0.60–0.90)^8^. Similar results were obtained from a retrospective cohort study involving 33,221 patients with newly diagnosed rheumatoid arthritis and 132,884 randomly selected age- and sex-matched patients without rheumatoid arthritis (HR: 0.65, 95% CI: 0.58–0.73)^9^. The heterogeneity of these results may be due to the inevitable confounding factors of observational studies. To clarify this relationship, we performed a two-sample MR analysis using genetic variants as instrumental variables, which can minimize the possibility of bias inherent to observational studies. As a result, our study provides novel and robust evidence for an inverse association between rheumatoid arthritis and the risk of PD.

Although our study provides evidence that patients with rheumatoid arthritis have a reduced risk of developing PD, the mechanisms underlying the protective effect is largely unknown. Our current finding together with earlier results^8,9,12^ contradicts the previous hypothesis that prolonged chronic inflammation and overproduction of inflammatory mediators in autoimmune diseases may activate microglia and contribute to the degeneration of neurons, thus increase the risk of developing PD^8,13^. The benefit of decreased risk for developing PD by rheumatoid arthritis cannot be attributed to therapeutic factors such as anti-inflammatory agent administration in rheumatoid arthritis either^8^, as MR approach elucidates the link using genetic instrumental variants. From another perspective, the activated microglia in the brain of PD patients may be a compensated consequence of the disease, and thus may be mainly associated with the progression of the disease, rather than initiation of the pathogenesis^14^. Further longitudinal observations of the progression rate of parkinsonism in patients with rheumatoid arthritis co-existing with PD may give credence to the viewpoint.

Notably, growing evidence implicates the role of lysosomal dysfunction in both autoimmune disorders and neurodegenerative diseases^15^. In the context of PD, the decrease of lysosomal enzyme activity, such as resulting from the mutation of *GBA* and *LRRK2*^2,16^, may lead to the aggregation of α-­synuclein and the formation of Lewy bodies, which is the pathological hallmark of PD^17^. Moreover, overexpression of lysosomal proteases cathepsin D could reduce the accumulation of α-­synuclein aggregates in mice model^18^. Strikingly, elevated levels of lysosomal enzyme activity have been reported to occur in several autoimmune diseases, including rheumatoid arthritis^15,19^. In rheumatoid arthritis, high levels of several lysosomal cathepsins (B, D, G, K, L, and S) are present both in the serum and synovial fluid of patients, which is contradictory to the condition of PD with a lower level of lysosomal enzyme activity^20^. Therefore, rheumatoid arthritis may shed the protective effect on PD through the lysosome pathway, which is worth further exploration. In addition, all treatments available for PD are symptomatic only and could not slow down the progression up till now. Therefore, it’s urgent and important to search for new effective therapeutic approaches for PD. The present results suggested that explorations of the mechanisms of inflammation and immune response, as well as lysosomal dysfunction, have the potential to provide new targets for the treatment of PD.

There were also some limitations to the current study. We excluded SNPs that were associated with smoking and BMI, but there are still other potential confounders that could violate the independence assumption, and it’s not practicable to rule out all of them. Moreover, population stratification and potential sample overlap might be another source of bias, as in all MR analyses. However, the F statistics calculated in the sensitivity analysis were all large enough, suggesting that the bias might be minimal. Meanwhile, the GWAS used in the MR analysis was conducted based on participants of European ancestry, thus the findings might be biased and not applicable to other populations. Future studies in other non-European populations will provide a more comprehensive understandings. In addition, the effect size identified in our study was not that large as observed in previous cohort studies. Future exploration based on summary data from GWAS with a larger sample size was warranted to provide a more accurate estimate.

In conclusion, based on the MR analysis results obtained from large-scale GWAS summary data, we demonstrate that rheumatoid arthritis could reduce the risk of PD in the European-ancestry population. Further exploration of the molecular mechanisms underlying the crosstalk between peripheral and central immunity may shed light on the understanding of the protective effect of rheumatoid arthritis on PD and reveal therapeutic targets for PD.

## Methods

### Datasets

We obtained summarized association results for rheumatoid arthritis from genome-wide association study (GWAS) on rheumatoid arthritis of European ancestry (*N*_case_ = 13,838, *N*_control_ = 33,742)^21^. Details of the summary data were listed in Supplementary Table 1, and the study design like the collection of samples, quality control procedures and imputation methods have been described in the original publication. We selected genetic variants that passed the genome-wide significance threshold (*P* < 5E–08) as instrument variants. Since smoking and body mass index (BMI) are associated with risk of both rheumatoid arthritis and PD, we removed SNPs which have been reported to be suggestively associated with these two traits by GWAS searched in GWAS Catalog^22^ (*P* < 1E–05). Then we clumped the instrument variables based on the 1000 Genomes Project linkage disequilibrium (LD) structure, and kept index SNPs with the lowest *P* value (*R*^2^ < 0.001 with any other associated SNP within 10 Mb). The summary statistics for the retained instrument variables were shown in Supplementary Table 2. As a replication, we also extracted instrumental variables from another GWAS on rheumatoid arthritis of European ancestry (*N*_case_ = 5539, *N*_control_ = 20,169)^23^ using the method mentioned above. The detailed summary data was shown in Supplementary Table 3.

Summary statistics of PD risk for the selected instrument variables were obtained from the published PD GWAS of European ancestry with the largest sample size so far (*N*_case_ = 33,647, *N*_control_ = 449,056)^24^. The study design like the collection of samples, quality control procedures, and imputation methods have been described in the original publication. We carried out harmonization to rule out strand mismatches and ensure the SNP effect sizes on rheumatoid arthritis and PD correspond to the same allele. The summary statistics were shown in Supplementary Tables 2, 3. This study only utilized summarized results from published studies, and individual data were not involved.

### Genetic correlation

We estimated the genetic correlation between PD and rheumatoid arthritis using GNOVA^25^ with default parameters. GNOVA estimates genetic covariance with the genetic variants summary data shared between two GWAS, and then calculates the genetic correlation based on genetic covariance and variant-based heritabilities. We ran GNOVA on all the SNPs in both diseases^24,26^ together with reference data derived from the 1000 Genomes Project European population.

### Mendelian randomization analysis

We hypothesized that rheumatoid arthritis as a risk factor could decrease the risk of PD, and used the MR approach to validate the hypothesis. The success of MR analysis depends on three assumptions: the genetic instruments are associated with risk of rheumatoid arthritis; the genetic instruments are not associated with potential confounders; the genetic instruments are associated with PD through the risk of rheumatoid arthritis (namely horizontal pleiotropy should not be present)^20^ (Supplementary Fig. 1).

We performed a two-sample MR analysis to estimate the effect of rheumatoid arthritis on PD by applying the fixed-effect inverse variance weighted (IVW) method, which is most widely used in MR studies and could provide robust causal estimates under the absence of directional pleiotropy. Meanwhile, we validated the results by applying MR–Egger regression, weighted mode, weighted median, and simple median methods in the discovery phase. We also performed MR analysis excluding variants within the extended major histocompatibility complex (MHC) region, since its complex LD structure made it susceptible to horizontal pleiotropy. In addition, to evaluate potential violations of the model assumptions in the MR analysis, we conducted extensive sensitivity analyses. Cochran’s Q test was computed to check heterogeneity across the individual causal effects. Mendelian Randomization Pleiotropy RESidual Sum and Outlier (MR-PRESSO) analysis was conducted to detect outlier instrument^27^. MR–Egger regression was performed to evaluate the directional pleiotropy of instruments. To evaluate the strongness of each instrument variable, we computed the F-statistic of each SNP as described by a previous study^28^. The leave-one-out analysis was conducted with the inverse variance weighted method to assess the influence of individual variants on the observed association. And we performed Steiger analysis^29^ to explore whether PD has a reverse causal impact on the risk of rheumatoid arthritis. The statistical power calculated at http://cnsgenomics.com/shiny/mRnd/ is sufficient (0.81) assuming the true causal OR of rheumatoid arthritis on PD is 0.9, given the involved sample size and the significance level α as 0.05^30^. The main statistical analyses were conducted using R package TwoSampleMR v0.5.5^31^.

### Reporting summary

Further information on research design is available in the Nature Research Reporting Summary linked to this article.

## Supplementary information

Supplementary Material

Reporting summary

## Data Availability

All data generated or analyzed during this study are included in this published article and its supplementary information files. Summary statistics of risk of PD was downloaded from IPDGC (https://pdgenetics.org/resources). Participants from 23andMe Inc. were excluded from the summary data.

## Code availability

Code or algorithm used to generate results in this study are available from the corresponding authors on reasonable request. Exposure instrumental variables could be obtained using functions in R package TwoSampleMR.
