# Supplementary material for: Rheumatoid arthritis decreases risk for Parkinson’s disease: a Mendelian randomization study
Source: NPJ Parkinsons Dis. 2021 Mar 2;7:17. doi: 10.1038/s41531-021-00166-x (PMC7925660; doi:10.1038/s41531-021-00166-x)
Supplement: Supplementary file 1 — Supplementary Material [file 41531_2021_166_MOESM1_ESM.pdf]

Rheumatoid Arthritis Decreases Risk for Parkinson's Disease: a Mendelian Randomization Study

Running head: Rheumatoid arthritis and PD

ChunYu Li[1], Ruwei Ou[1], HuiFang Shang[1*]

[1]Department of Neurology, Laboratory of Neurodegenerative Disorders, National Clinical Research Center for Geriatric, West China Hospital, Sichuan University, Chengdu 610041, China.

*Corresponding author: HuiFang Shang

Affiliation: Department of Neurology, Laboratory of Neurodegenerative Disorders, National Clinical Research Center for Geriatric, West China Hospital, Sichuan University, Chengdu 610041, China

Postal address: No.37, Guoxue Lane, 610041, Chengdu, Sichuan, China

E-mail: hfshang2002@126.com

Telephone Number: 0086-18980602127

Supplementary Figure 1. Assumptions in Mendelian randomization analysis.

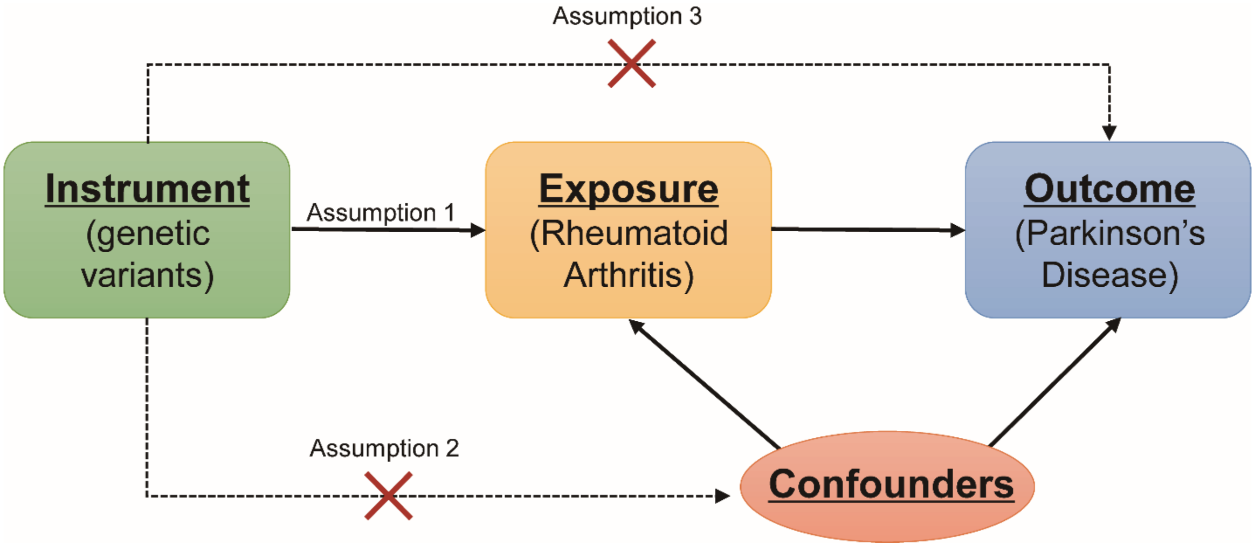

Broken lines represent potential pleiotropic or direct causal effects between variables that would violate Mendelian randomization assumptions. Assumption 1: Genetic variants are associated with the rheumatoid arthritis; Assumption 2: Genetic variants are not associated with confounders like smoking and body mass index; Assumption 3: Genetic variants influence the outcome only through the exposure

Supplementary Table 1. Summary data from all GWAS used in current study

Disease	Cases	Controls	Ethnics	SNPs	Pubmed ID
discovery rheumatoid arthritis	13,838	33,742	EUR	112,654	23143596
replication rheumatoid arthritis	5,539	20,169	EUR	2,556,272	20453842
Parkinson's disease	33,647	449,056	EUR	17,510,617	31701892

EUR, European; SNP, single nucleotide polymorphism; GWAS, genome-wide association study; PD summary statistics were from Nalls et al 2019 excluding participants from 23andMe Inc.

Supplementary Table 2. Summary information for instrumental variables of rheumatoid arthritis in discovery phase

SNP	Genomic position (GRCh37)	Gene	alleles	rheumatoid arthritis			Parkinson's disease			F statistics
				P value	Beta	SE	P value	Beta	SE	
rs6679677	1:114303808	PHTF1	A/C	1.23E-61	0.466	0.028	0.197	-0.038	0.029	274.43
rs2228145	1:154426970	IL6R	C/A	1.32E-08	-0.109	0.019	0.261	0.019	0.017	32.31
rs2240339	1:17674108	PADI4	T/C	1.36E-08	-0.112	0.020	0.860	0.003	0.018	32.25
rs8026898	15:69991417	PCAT29	A/G	2.22E-09	0.125	0.021	0.928	-0.002	0.019	35.77
rs10209110	2:100672692	AFF3	T/C	1.13E-08	-0.106	0.019	0.069	0.031	0.017	32.61
rs13426947	2:191933254	STAT4	A/G	7.19E-10	0.143	0.023	0.947	-0.001	0.021	37.97
rs17630466	4:26086569	LINC02357	G/A	2.45E-10	0.127	0.020	0.921	0.002	0.020	40.07
rs71624119	5:55440730	ANKRD55	A/G	5.59E-20	-0.207	0.023	0.761	-0.006	0.021	83.76
rs6920220	6:138006504	intergenic	A/G	1.84E-10	0.141	0.022	0.090	-0.035	0.021	40.63
rs9262218	6:30797215	LINC00243	T/C	1.22E-10	-0.447	0.069	0.696	0.018	0.045	41.43
rs3129886	6:32410576	HLA-DRA	C/T	3.20E-79	0.441	0.023	0.817	-0.005	0.022	355.15
rs660895	6:32577380	intergenic	G/A	1.00E-200	0.818	0.021	5.80E-07	-0.131	0.026	1479.74
rs3129294	6:33084671	HLA-DPB2	C/A	6.96E-19	-0.177	0.020	0.499	0.015	0.022	78.78
rs2812378	9:34710260	CCL21	A/G	1.31E-08	-0.110	0.019	0.212	0.026	0.021	32.32

SNP: single nucleotide polymorphism, beta: SNP effect size, SE: standard error of the SNP effect size.

All the selected instruments together explain about 2.51% phenotypic variation of rheumatoid arthritis risk at the observed scale.

Supplementary Table 3. Summary information for instrumental variables of rheumatoid arthritis in replication phase

SNP	Genomic position (GRCh37)	Gene	alleles	rheumatoid arthritis			Parkinson's disease			F statistics
				P value	Beta	SE	P value	Beta	SE	
rs6679677	1:114303808	PHTF1	A/C	4.39E-70	0.663	0.038	0.197	-0.038	0.029	302.43
rs3087243	2:204738919	CTLA4	A/G	2.24E-08	-0.139	0.023	0.083	0.029	0.017	35.20
rs4810485	20:44747947	CD40	G/T	5.69E-09	0.163	0.030	0.386	-0.017	0.019	29.26
rs10040327	5:55435220	ANKRD55	A/C	6.65E-11	-0.288	0.044	0.475	-0.022	0.030	42.68
rs6920220	6:138006504	intergenic	A/G	2.49E-12	0.199	0.029	0.090	-0.035	0.021	46.04
rs9268145	6:32257284	TSBP1-AS1	G/T	1.00E-200	1.058	0.027	2.52E-05	-0.107	0.025	1581.71
rs9784876	6:32788878	TAP2	A/C	2.40E-46	0.668	0.047	0.833	0.008	0.037	202.00
rs460568	6:33232025	VPS52	T/C	3.81E-16	0.262	0.031	0.477	0.017	0.024	69.65

SNP: single nucleotide polymorphism, beta: SNP effect size, SE: standard error of the SNP effect size.

All the selected instruments together explain about 2.04% phenotypic variation of rheumatoid arthritis risk at the observed scale.
